# Moxibustion Protects Dopaminergic Neurons in Parkinson's Disease through Antiferroptosis

**DOI:** 10.1155/2021/6668249

**Published:** 2021-04-16

**Authors:** Zifeng Huang, Wenwen Si, Xinrong Li, Shanyu Ye, Xuelei Liu, Yichun Ji, Xiaoqian Hao, Dongfeng Chen, Meiling Zhu

**Affiliations:** ^1^Traditional Chinese Medicine Innovation Research Center, Shenzhen Hospital of Integrated Traditional Chinese and Western Medicine, Shenzhen, Guangdong 518104, China; ^2^Graduate School, Guangzhou University of Chinese Medicine, Guangzhou, Guangdong 510405, China; ^3^Department of Anatomy, The Research Center of Basic Integrative Medicine, Guangzhou University of Chinese Medicine, Guangzhou, China; ^4^Shenzhen Bao An Traditional Chinese Medicine Hospital, Guangzhou University of Chinese Medicine, Shenzhen, China

## Abstract

Ferroptosis is associated with neural degeneration of dopaminergic neurons in Parkinson's disease (PD). However, how to control the level of ferroptosis in PD remains unclear. Clinically, moxibustion has been used to treat PD and has an apparent therapeutic effect on improving the motor symptoms of PD. In the present study, the PD rat model was constructed by two-point stereotactic 6-hydroxydopamine injection. Then, moxibustion was used to treat the PD rats. The expression of glutathione peroxidase 4 (GPX4) and Ferritin Heavy Chain 1 (FTH1), the level of reactive oxygen species (ROS), and the morphology of mitochondrial were detected to evaluate the level of ferroptosis. The results showed that moxibustion treatment of Shi's moxa sticks could reduce the behavioral score, alleviate the level of ferroptosis, decrease mitochondrial damage, and improve dopaminergic neuron survival. In conclusion, the present study results indicated that Shi's moxa sticks could effectively suppress the level of ferroptosis, thereby improving the survival of dopaminergic neurons in the SNpc of PD rats, which may provide a promising complementary and alternative therapy for PD patients.

## 1. Introduction

Parkinson's disease (PD) is a degenerative disease of the aging people's nervous system. It has become the second primary neurodegenerative disease worldwide [1]. It is estimated that the prevalence rate of PD is about 2% in the population over 50 years of age and 2.5% in the population over 70 years of age world [2]. PD is primarily characterized by the degeneration and death of the dopaminergic neurons in the SNpc [3]. PD's motor symptoms, such as resting tremor, bradykinesia, and rigidity, can severely affect the patient's daytime function and quality of life [4]. Furthermore, patients' gradual deterioration leads to higher disability rates, which will pose a sizeable economic burden to the family and society [5]. At present, the primary treatment for PD is levodopa, which can significantly reduce PD symptoms [6]. However, motor complications caused by levodopa's long-term use are a significant drawback of levodopa in PD treatment [7]. Thus, there is an urgent need to search for effective treatment of PD.

Moxibustion is a form of external therapy in traditional Chinese medicine [8]. Recent research suggested that moxibustion treatment can postpone PD progression and has an apparent therapeutic effect on motor symptom improvement in PD patients [9, 10]. Moreover, numerous animal experiments have confirmed that moxibustion can alleviate PD injury. The mechanism may be related to the protection of dopaminergic neurons in SNpc [11, 12]. In the present study, we investigated the protective effects of Shi's moxa stick on PD rat's dopaminergic neuron injury. Shi's moxa stick was developed by Shi Xuemin, an academician of the Chinese Academy of Engineering and a master of traditional Chinese medicine, and has been used in our hospital to relieve PD symptoms.

There is increasing evidence that ferroptosis is associated with the occurrence of PD [13]. Ferroptosis is a novel form of nonapoptotic programmed cell death closely related to iron metabolism disturbance. It is closely associated with various neurodegenerative diseases, including PD [14–16]. The occurrence of ferroptosis is related to a decrease of glutathione peroxidase 4 (GPX4) and Ferritin Heavy Chain 1 (FTH1) [17], an increase of reactive oxygen species (ROS) [18], and the damage of mitochondrial [18–20]. GPX4 is a type of antioxidase that can reduce intracellular lipid peroxide levels and avoid oxidative damage. The decrease of GPX4 activity will lead to ROS accumulation, which will eventually induce ferroptosis [21]. FTH1 is a major iron storage protein and maintains intracellular iron balance [22]. The excess iron may lead to ferroptosis in the cells [23]. Our previous studies have shown that the pathology mechanisms of PD were related to the level of ferroptosis, which can be regulated by FTH1 [17, 24]. Observation of mitochondrial morphology and size by transmission electron microscope is an essential indicator of ferroptosis. The morphological changes of ferroptosis include reducing mitochondrial volume, an increase of double-layer membrane density, and reduction or disappearance of mitochondrial cristae [18, 25]. These indicators will be used to detect the level of ferroptosis and explore how to suppress ferroptosis in PD.

In this study, we investigated the specific mechanism of moxibustion in improving symptoms of PD. Our results confirmed that moxibustion could effectively suppress the level of ferroptosis, thereby improving the survival of dopaminergic neurons in the SNpc of PD rats, which may provide a promising complementary and alternative therapy for PD patients.

## 2. Materials and Methods

### 2.1. Animals

A total of 108 male Sprague-Dawley (SD; 180–200 g; 8-9 weeks old) rats were purchased from Guangzhou Dean Gene Technology Co., Ltd., and were placed under standard laboratory conditions (21–25°C, 50–70% relative humidity, 12-hour light-dark cycle, and food and water ad libitum). All rats were allowed to acclimate for one week before PD surgery. All animal procedures followed the rules of the National Institute of Health Guide for the Care and Use of Laboratory Animals.

### 2.2. Experiment Protocol

#### 2.2.1. Experiment 1

To observe the degeneration of dopaminergic neurons and the occurrence of ferroptosis in the PD rat model, the rats were randomly divided into three groups: control group, sham group, and 6-OHDA group.

#### 2.2.2. Experiment 2

To validate the efficacy of moxibustion treatments in PD, the rats were randomly divided into four groups: control group, sham group, 6-OHDA group, and moxibustion group. When the behavioral test was complete, the rats (*n* = 18/group) were anesthetized and sacrificed, with the fresh right side of the brains being quickly removed to −80°C (*n* = 6/group). At the same time, the other was fixed using 4% paraformaldehyde solution (*n* = 6/group) and 2.5% glutaraldehyde (*n* = 6/group).

### 2.3. PD Rat Model Preparation

The PD rat model was established using a two-point 6-hydroxydopamine injection in the right SN and ventral tegmental area [24]. The rats used in the experiments were under anesthesia (100 mg/kg ketamine and 10 mg/kg xylazine, intraperitoneal injection). In the PD model rats, the rat's head was shaved to expose the scalp and fixed on the stereotactic frame. 4 *μ*l 6-OHDA (cat. no. H116-5 mg; Sigma-Aldrich; use 0.02% ascorbate solution to dissolve to 8 *μ*g/*μ*l) was intracerebroventricularly injected into two sites (AP: −4.9 mm, L: −1.9 mm, and DV: −7.5 mm and AP: −4.9 mm, L: −1.1 mm, DV: 8.0 mm relative to the bregma and dural surface) by 5 *μ*l microsyringe. After injection, we left the needle in the brain for 10 minutes and then withdrew the needle at a rate of 1 mm/min. Finally, we put a piece of gelatin sponge into the burr holes to prevent bleeding and cerebrospinal fluid leakage and suture the skin wound. The rat's body temperature was kept at 36.5°C with a heating pad during the procedure until the rat recovers from anesthesia. The same procedures were performed in the sham group, but 4 *μ*l of saline was administered that contained 0.02% ascorbic acid instead of 6-OHDA.

### 2.4. Moxibustion Treatment of Shi's Moxa Stick

After four weeks of 6-OHDA lesioning, rats in the moxibustion group were treated with Shi's moxa sticks after model establishment. The current study chose Baihui (GV20) acupoint. For the moxa stick to reach the acupoint, the area of Baihui on the rat's head was shaved to expose the skin at the beginning of treatment. The moxibustion treatment of Shi's moxa sticks lasts for 30 minutes per day, six times a week, for four weeks. The rats in the control group, the sham group, and the model group received normal feeding and did not undergo any treatment for four weeks.

### 2.5. Behavioral Test

Before collecting tissue, all rats were injected with intraperitoneal apomorphine (APO, cat. no. 017–18321; Wako; 0.5 mg/kg) for behavioral test. Ten minutes after APO injection, the rat started to spin and the number of complete turns (360°) to the left side for 30 min was recorded.

### 2.6. Immunohistochemical Staining

To observe the expression of TH in the substantia nigra, six rats in each group were in under anesthesia. Firstly, we used normal cold saline for blood washing through the cardiac aorta injection and then fixed the brain tissue with 4% paraformaldehyde solution. After fixation, the brain tissue was gently removed, placed in a 4% paraformaldehyde solution, and sectioned into 10 *μ*m thick coronal slices. Frozen sections (10 *μ*m thickness) of the brain were permeabilized in 0.3% Triton X-100 solution for 30 min. Then, we performed immunohistochemical staining with Histostain-Plus Kits (cat. no. SP-0022; Bioss) according to the manufacturer's protocol. Primary antibody against TH (1 : 1000; cat. no. ab152; Millipore) was incubated at 4°C overnight. After washing with PBS, diaminobenzidine (DAB) working solution (cat. no. C02-04001; Bioss) was added for color rendering. Each section was restained by hematoxylin (cat. no. DH0005; Mayer), dehydrated, and fixed with ethanol according to the concentration gradient. The histopathological observations were documented by light microscopy at 400x magnification, and images were captured. We repeated each experiment three times, and data were expressed as the mean ± SEM.

### 2.7. Hematoxylin/Eosin (HE) Staining

To observe neurons' morphology in the substantia nigra, frozen sections (10 *μ*m thickness) were used for HE staining. HE staining was performed according to the standard protocol, and histopathological observation results were recorded by light microscopy at 400x magnification, and images were captured.

### 2.8. ROS Detection of Articular Cartilage

To observe the level of ROS, fresh SN tissue from each group was made into homogenate with buffer. Then, we performed ROS detection with BBoxiProbe O13 (cat. no. BB-470512; BestBio) according to the manufacturer's protocol. We repeated each experiment three times, and data were expressed as the mean ± SEM.

### 2.9. Transmission Electron Microscope

A transmission electron microscope was used to observe the morphology of mitochondria. Six rats in each group were anesthetized. Through the injection of the cardiac aorta, we used normal cold saline for blood washing at first and then fixed the brain tissue with 2.5% glutaraldehyde solution (cat. no. DF0156; Leagene) at 4°C. We cut out the SN tissue, fixed it with 1% osmium acid, and washed it 3 times with 0.1 M phosphate buffer. The tissue was then fixed with 1% osmium tetroxide and dehydrated through an alcohol gradient before being embedded in resin. The sample was detected by transmission electron microscopy.

### 2.10. Western Blot Analysis

The protein was extracted from the SNpc tissue by RIPA lysis buffer (cat. no. R0278; Sigma-Aldrich). After centrifugation at 12,000 rpm for 20 min at 4°C, total proteins were harvested. The bicinchoninic acid assay (BCA; cat. no. 23225; Thermo Fisher Scientiﬁc) was used to determine the concentration of total proteins. The total protein (30 ug) was separated with 10% SDS-PAGE gels and transferred to polyvinylidene difluoride membranes (PVDF; Millipore). After blocking with 5% BSA for 2 h, the membrane was incubated with primary antibodies (TH, 1 : 1000, cat. no. ab152, Millipore; GPX4, 1 : 1000, cat. no. ab125066, Abcam; FTH1, 1 : 1000, cat. no. ab183781, Abcam; GAPDH, 1 : 5000, cat. no. ab8245, Abcam) at 4°C overnight. The membrane was then incubated with HRP-labelled secondary antibodies (horseradish peroxidase- (HRP-) conjugated goat anti-rabbit, 1 : 5000, cat. no ab6721, Abcam; HRP-conjugated goat anti-mouse, 1 : 5000, cat. no. ab6789, Abcam) for 1 hour at room temperature. The ECL substrate (Pierce, Thermo Fisher Scientific, Inc. USA) was used to visualize the bands. The data were analyzed by image *j*.

### 2.11. Extraction of Total RNA and RT-qPCR

Total RNA from the SNpc was extracted and purified using the Direct-zol RNA Kit (cat. no. R2070; ZYMO research) according to the manufacturer's protocol, and the RNA was stored at −80°C. Reverse transcription and qPCR assays were performed using the PrimeScript RT reagent kit (Cat.no. RR600 A; Takara) and the TB Green Premix Ex Taq II (Cat. no. RR820 A; Takara) on Light Cycler 480 SYBR Green I Master (Roche Diagnostics, GmbH, Mannheim, Germany). Each experiment was replicated three times, and data are presented as the mean ± SEM. Primers are shown as follows:

  TH forward: 5′-ATTGCCTTCCAGTACAAGCAC-3′;
  TH reverse: 5′-CCTTCAGCGTGACATATACCTCC-3′;
  GPX4 forward: 5′-ATAAGAACGGCTGCGTGGTGAAG-3′;
  GPX4 reverse: 5′-TAGAGATAGCACGGCAGGTCCTTC-3′;
  FTH1 forward: 5′-TTCAGGGCCACATCATCCCG-3';
  FTH1 reverse: 5′-GCAAGTGCGCCAGAACTACC-3′;
 
*β*-acting forward: 5′-CTCAGGAGAGGAGCCATTTATT-3′;
 
*β*-acting reverse: 5′-CCCGATCAGAGTGAAGCTATT-3′.

### 2.12. Statistical Analyses

The experimental data are presented as the mean ± SEM. SPSS 22.0 software (IBM Corp., Armonk, NY, USA) was used for statistical analysis. The figures were all produced using GraphPad Prism (Version 6.0; GraphPad Software, Inc., La Jolla, CA, USA). A two-tailed unpaired *t*-test was used to analyze the difference between the two groups. The comparison between multiple groups was analyzed using a one-way analysis of variance followed by Tukey's multiple comparisons test as the post hoc test. *P* < 0.05 was considered to indicate a statistically significant difference.

## 3. Result

### 3.1. The PD Rats Model Exhibits Degeneration of Dopaminergic Neurons

**Figure 1:**
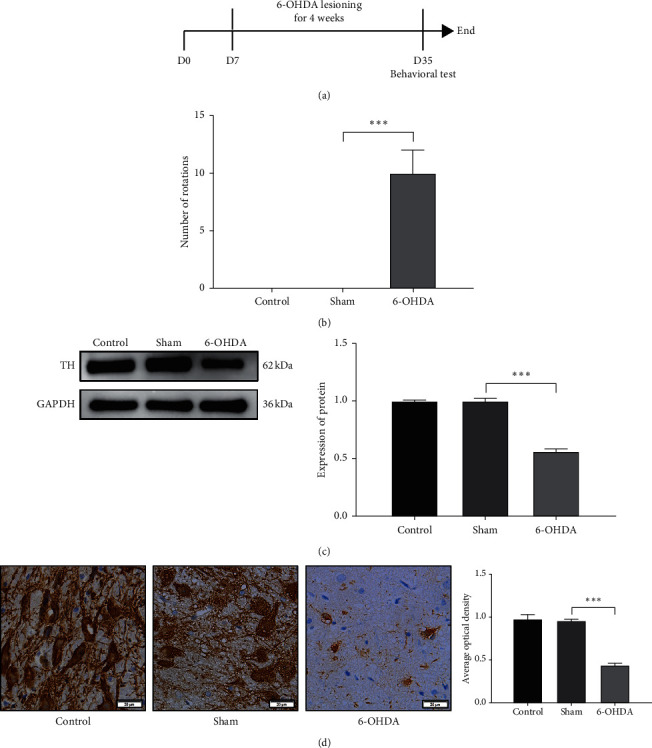
The behavioral score of PD rats increased, and the protein expression level of TH decreased. (a) The timeline of animal experiment 1. (b) The behavioral score of the control group, the sham group, and the 6-OHDA group. (c) TH level of total protein in SNpc of the control group, the sham group, and the 6-OHDA group. (d) IHC showed the expression of TH in the substantia nigra of the control group, the sham group, and the 6-OHDA group.

Behavioral test was applied to explore whether the PD rats model was successfully constructed after four weeks of two-point stereotactic 6-hydroxydopamine injection [26] (Figure 1(a)). The behavioral test revealed that the behavioral score increased significantly after stereotactic injection, and no animal in the normal group or sham group exhibited rotating behavior (Figure 1(b); sham versus 6-OHDA; *P* < 0.001). The expression of TH is a marker of dopaminergic neurons [27] and reflects the activity of dopaminergic neurons. The Western blot and immunohistochemistry showed that the expression of TH was significantly decreased in the 6-OHDA group compared with the sham group (Figure 1(c); sham versus 6-OHDA; *P* < 0.001; Figure 1(d); sham versus 6-OHDA; *P* < 0.001). These results showed that the PD rat model was successfully constructed, and the survival of dopaminergic neurons was decreased.

### 3.2. The PD Rats Model Exhibits an Increased Level of Ferroptosis

**Figure 2:**
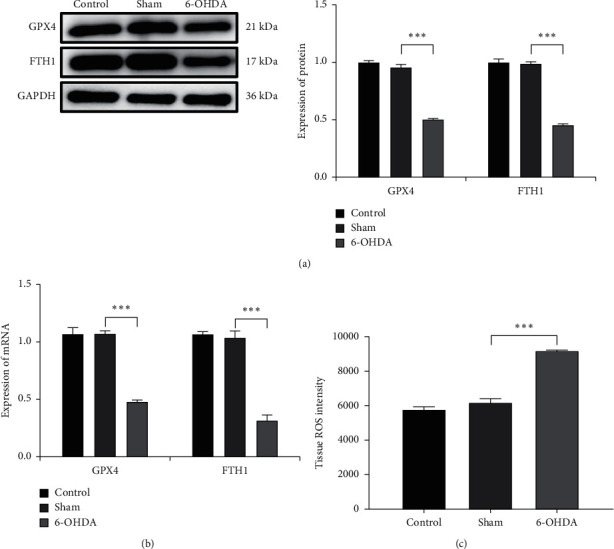
The level of ferroptosis in PD rats is increased. (a) GPX4 and FTH1 level of total protein in the control group, the sham group, and the 6-OHDA group. (b) GPX4 and FTH1 levels of total mRNA in the control group, the sham group, and the 6-OHDA group. (c) The level of ROS in the control group, the sham group, and the 6-OHDA group.

To evaluate the level of ferroptosis in the PD rats model, we detected the expression of GPX4, FTH1, and ROS. The Western blot showed that the expression of GPX4 and FTH1 was significantly decreasing in the 6-OHDA group compared with the sham group (Figure 2(a); sham versus 6-OHDA; *P* < 0.001). A similar trend was observed for the GPX4 and FTH1 expression levels of mRNA (Figure 2(b); sham versus 6-OHDA; *P* < 0.001). The occurrence of ferroptosis is associated with the accumulate of ROS. The result showed that the level of ROS was increased obviously in the 6-OHDA group (Figure 2(c); sham versus 6-OHDA; *P* < 0.001). These data suggested that the level of ferroptosis in PD rats is increased.

### 3.3. Moxibustion Treatment of Shi's Moxa Stick Has a Positive Effect on Suppressing the Death of Dopaminergic Neurons in the PD Rats Model

**Figure 3:**
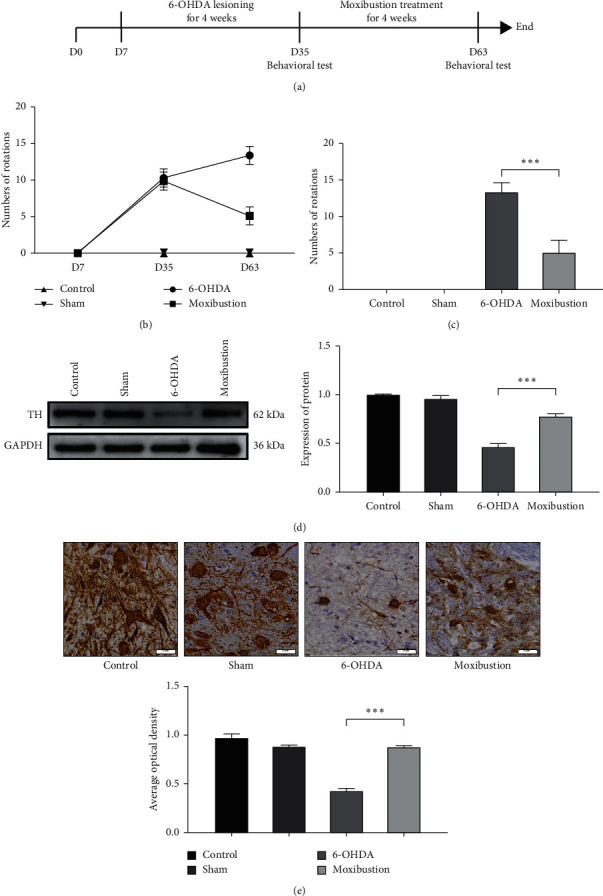
Moxibustion treatment of Shi's moxa stick has a positive effect on suppressing the death of dopaminergic neurons in the PD rats model. (a) The timeline of animal experiment 2. (b) The change of behavioral score in D35 and D63 of the control group, the sham group, the 6-OHDA group, and the moxibustion group. (c) The behavioral score in D63 of the control group, the sham group, the 6-OHDA group, and the moxibustion group. (d) TH level of total protein in SNpc of the control group, the sham group, the 6-OHDA group, and the moxibustion group. (e) IHC showed the expression of TH in the substantia nigra of the control group, the sham group, the 6-OHDA group, and the moxibustion group.

**Figure 4:**
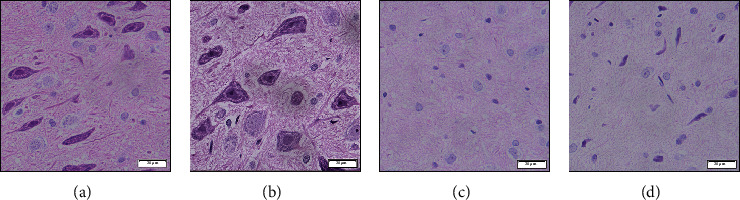
Moxibustion treatment of Shi's moxa stick positively affects neurons' morphology in the PD rats model. HE staining was performed in the SN of the control group, the sham group, the 6-OHDA group, and the moxibustion group. (a) Control. (b) Sham. (c) 6-OHDA. (d) Moxibustion.

To assess whether moxibustion could alleviate the injury of dopamine neurons in PD, we performed two-point stereotactic 6-hydroxydopamine injection on rats again. After the PD rats' model was established, moxibustion was performed on the PD rats with Shi's moxa stick for 30 minutes per day, six times a week, for four weeks (Figure 3(a)). To evaluate whether the moxibustion treatment of Shi's moxa stick could improve the survival of dopaminergic neurons, we detected the behavioral test, the expression of TH, and the morphology of neurons. As a show in Figure 3(b), we detected that the score of behavioral test was still increased after D35 in the 6-OHDA group, which demonstrates that also the PD model was established and the 6-OHDA was still prompt neuronal death. In contrast, the behavioral score was significantly decreased after moxibustion treatment of Shi's moxa sticks in the moxibustion group. And the moxibustion group's behavioral score was decreased significantly compared with the 6-OHDA group (Figure 3(c); 6-OHDA versus moxibustion; *P* < 0.001). According to the result of western blotting, the expression of TH in the moxibustion group was significantly increased compared with the 6-OHDA group (Figure 3(d); 6-OHDA versus moxibustion; *P* < 0.001). A similar trend was confirmed by the results of immunohistochemistry (Figure 3(e); 6-OHDA versus moxibustion; *P* < 0.001). The result showed that, after moxibustion treatment of Shi's moxa stick, the activity of dopaminergic neurons was increased According to HE staining (Figure 4), the shape of the nerve cells in the sham group was normal. However, in the 6-OHDA group, the shape of neurons was rounded, and the nucleus was concentrated or swollen. After moxibustion treatment of Shi's moxa sticks, although some never cells still have abnormal morphology, the number of nerve cells with healthy morphology was increased. This proved that moxibustion treatment of Shi's moxa stick could alleviate the injury of dopamine neurons in PD.

### 3.4. Moxibustion Treatment of Shi's Moxa Stick Suppresses the Level of Ferroptosis in the PD Rats Model

**Figure 5:**
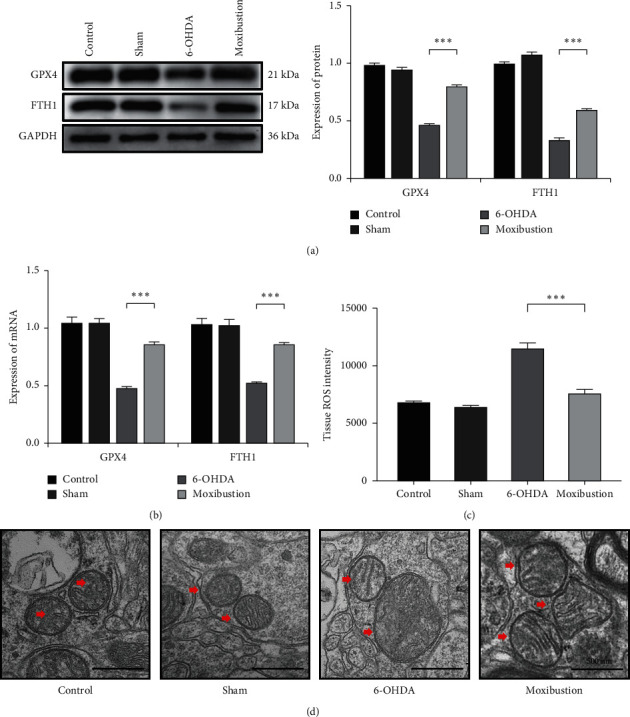
After moxibustion treatment of Shi's moxa stick, the level of ferroptosis is decreased. (a) GPX4 and FTH1 level of total protein in the control group, the sham group, the 6-OHDA group, and the moxibustion group. (b) GPX4 and FTH1 levels of total mRNA in the control group, the sham group, the 6-OHDA group, and the moxibustion group. (c) The level of ROS in the control group, the sham group, the 6-OHDA group, and the moxibustion group. (d) Observe the morphology of mitochondrial in the control group, the sham group, the 6-OHDA group, and the moxibustion group. The red arrow refers to mitochondria.

To investigate whether the moxibustion treatment of Shi's moxa stick can suppress the level of ferroptosis in the PD rats model, the expression of GPX4, FTH1, and ROS was used. The Western blot and Rt-PCR analysis results showed that GPX4 and FTH1 expressions were increased significantly after moxibustion treatment of Shi's moxa stick (Figure 5(a); 6-OHDA versus moxibustion; *P* < 0.001; Figure 5(b); 6-OHDA versus moxibustion; *P* < 0.001), whereas ROS was decreased obviously after moxibustion treatment of Shi's moxa stick (Figure 5(c); 6-OHDA versus moxibustion; *P* < 0.05). The morphological changes of ferroptosis are mainly the damage of mitochondria. As shown in Figure 5(d), in the 6-OHDA group, mitochondrial cristae ruptured or disappeared, and the structure was fuzzy. After moxibustion treatment of Shi's moxa stick, mitochondrial morphology damage is reduced. This proved that the protective effect of moxibustion treatment of Shi's moxa stick on dopaminergic neurons may be related to effectively suppressing the level of ferroptosis.

## 4. Discussion

As a progressive neurodegenerative movement disorder, there is an urgent need for an effective treatment for PD [1]. In this study, we hope to provide promising therapies for PD. The present study constructed the PD rat model by a two-point stereotactic injection of 6-hydroxydopamine and investigated moxibustion treatment's functional roles using Shi's moxa stick in PD. The present study's significant results were as follows: (i) the level of ferroptosis is associated with the injury of dopamine neurons in PD models; (ii) moxibustion treatment of Shi's moxa stick can alleviate the damage of dopamine neurons by suppressing ferroptosis. Our research provides a promising treatment and therapeutic target to improve the clinical outcome of PD treatment.

Research shows that abnormal iron metabolism and iron homeostasis are involved in PD's pathological process [14, 28]. Previous studies have shown that FTH1, GPX4, and ROS can be used as a marker to monitor the level of ferroptosis [22, 29]. In contrast, the morphology of ferroptosis mainly manifested the reduced mitochondrial volume, the increase of double-layer membrane density, and the reduction or disappearance of mitochondrial cristae [18, 25]. An essential finding of the present study is that GPX4 and FTH1 decrease significantly, and the level of ROS was increased obviously in the PD rats model. Simultaneously, directly observing the mitochondrial morphology of different groups through transmission electron microscopy, mitochondrial cristae ruptured or disappeared, and the structure was fuzzy in the PD rats model, representing high ferroptosis levels in the PD rat model. These results are consistent with a recent study, which found that ferroptosis plays a vital role in PD development [30]. This study shows that antiferroptosis of moxibustion treatment can be used as a protection pathway for PD patients.

Another important finding of this study is that moxibustion treatment of Shi's moxa stick can effectively suppress dopaminergic neurons' death. As a form of external therapy in traditional Chinese medicine, moxibustion treatment reported can ameliorate motor symptoms in PD patients [9, 10]. Shi's moxa sticks were developed by Shi Xuemin, an academician of the Chinese Academy of Engineering and a master of traditional Chinese medicine. Shi's moxa sticks have been used in hospitals to relieve PD symptoms. Baihui (GV20) is located at the midpoint of the two ears above the head. Because the head is the meeting point of Governor Vessel and all Yang meridians, Baihui (GV20) can regulate the yang qi of all Yang meridians [24]. Existing research proves that Baihui improves blood circulation in the brain and enhances memory [31]. Therefore, this study chose Baihui as an acupoint. The expression of TH is a crucial link of the DA neuron [32]. Our results show that, after moxibustion treatment of Shi's moxa stick, the rotational behavioral score of PD rats was decreased, the expression of TH, GPX4, and FTH1 was increased, the level of ROS was decreasing, and mitochondrial damage was relieved. However, in the 6-OHDA group, the symptoms of PD rats continued to worsen. As shown in the behavioral score, the behavioral score slightly increased in the D63 compared to the D35, which demonstrates that also PD model has been established in D35, and the 6-OHDA was still promoting neuronal death. This finding may indicate that moxibustion treatment of Shi's moxa stick can effectively suppress dopaminergic neurons' death, while the 6-OHDA still promotes neuronal death. The protective effect of moxibustion treatment may be related to the effective suppression of ferroptosis levels.

The limitation of this study is that the specific molecular mechanism of how moxibustion treatment of Shi's moxa sticks suppresses ferroptosis level by increasing the expression of FTH1 has not been explained in this study, which needs further research. In conclusion, we found that the moxibustion treatment of Shi's moxa sticks improves DA neurons' survival by suppressing the ferroptosis level. Moxibustion treatment of Shi's moxa stick is an economical, safe, and convenient way for PD treatment and a promising complementary and alternative therapy for PD patients.

## Data Availability

The data and materials produced during the study can be obtained from the corresponding authors upon reasonable request.
